# Mutations in *NKX6-2* Cause Progressive Spastic Ataxia and Hypomyelination

**DOI:** 10.1016/j.ajhg.2017.05.009

**Published:** 2017-06-01

**Authors:** Viorica Chelban, Nisha Patel, Jana Vandrovcova, M. Natalia Zanetti, David S. Lynch, Mina Ryten, Juan A. Botía, Oscar Bello, Eloise Tribollet, Stephanie Efthymiou, Indran Davagnanam, SYNAPSE Study Group, Fahad A. Bashiri, Nicholas W. Wood, James E. Rothman, Fowzan S. Alkuraya, Henry Houlden

**Affiliations:** 1 Department of Molecular Neuroscience, University College London, London WC1N 3BG, UK; 2 Developmental Genetics Unit, Department of Genetics, King Faisal Specialist Hospital and Research Center, MBC 03, PO Box 3354, Riyadh 11211 Saudi Arabia; 3 Department of Clinical and Experimental Epilepsy, University College London, London WC1N 3BG, UK; 4 Neurogenetics Laboratory, The National Hospital for Neurology and Neurosurgery, London WC1N 3BG, UK; 5 Department of Anatomy and Cell Biology, College of Medicine, Alfaisal University, Riyadh 11533, Saudi Arabia; 6 Department of Pediatrics, College of Medicine, King Saud University, Riyadh 11451, Saudi Arabia; 7 Department of Neurology and Neurosurgery, Institute of Emergency Medicine, Toma Ciorbă 1, 2052 Chisinau, Republic of Moldova; 8 Department of Brain Repair and Rehabilitation, University College London, London WC1N 3BG, UK; 9 Reta Lila Weston Research Laboratories, Institute of Neurology, University College London, London WC1N 3BG, UK; 10 Department of Information and Communications Engineering, University of Murcia, Campus Espinardo, 30100 Murcia, Spain; 11 Department of Medical and Molecular Genetics, King’s College London, Guy’s Hospital, SE1 9RT London, UK; 12 See Document S1 for list of collaborators; 13 Department of Cell Biology, Yale School of Medicine, New Haven, CT 06520-8002; 14 Saudi Human Genome Program, King Abdulaziz City for Science and Technology, Riyadh 12371, Saudi Arabia

**Keywords:** spasticity, ataxia, leukodystrophy, NKX6-2, recessive, genetic

## Abstract

Progressive limb spasticity and cerebellar ataxia are frequently found together in clinical practice and form a heterogeneous group of degenerative disorders that are classified either as pure spastic ataxia or as complex spastic ataxia with additional neurological signs. Inheritance is either autosomal dominant or autosomal recessive. Hypomyelinating features on MRI are sometimes seen with spastic ataxia, but this is usually mild in adults and severe and life limiting in children. We report seven individuals with an early-onset spastic-ataxia phenotype. The individuals come from three families of different ethnic backgrounds. Affected members of two families had childhood onset disease with very slow progression. They are still alive in their 30s and 40s and show predominant ataxia and cerebellar atrophy features on imaging. Affected members of the third family had a similar but earlier-onset presentation associated with brain hypomyelination. Using a combination of homozygozity mapping and exome sequencing, we mapped this phenotype to deleterious nonsense or homeobox domain missense mutations in *NKX6-2*. *NKX6-2* encodes a transcriptional repressor with early high general and late focused CNS expression. Deficiency of its mouse ortholog results in widespread hypomyelination in the brain and optic nerve, as well as in poor motor coordination in a pattern consistent with the observed human phenotype. In-silico analysis of human brain expression and network data provides evidence that *NKX6-2* is involved in oligodendrocyte maturation and might act within the same pathways of genes already associated with central hypomyelination. Our results support a non-redundant developmental role of *NKX6-2* in humans and imply that *NKX6-2* mutations should be considered in the differential diagnosis of spastic ataxia and hypomyelination.

## Main Text

The clinical combination of hereditary spastic paraplegia and cerebellar ataxia forms a frequent and heterogeneous group of neurological conditions. Progression varies and is often slow when affected individuals present in the adult neurology setting, whereas in children the condition is usually rapid and associated with other clinical features, such as epilepsy, cognitive decline, dystonia, and hypomyelination on MRI.

Early and widespread clinical signs reflect the pivotal position of myelination in the nervous system and the need for adequate and measured myelination for many critical functions^1^ and preservation of brain plasticity.^2^, ^3^ Defects in myelin underlie many neurological disorders, some acquired (e.g., immune-mediated destruction of myelin in multiple sclerosis) and some congenital (e.g., inborn errors of myelin metabolism). The latter group of hypomyelinating diseases is highly heterogeneous; the phenotype depends on the site of involvement (central nervous system versus peripheral nerves), severity, and age of onset. Hypomyelinating leukodystrophies are genetically determined diseases characterized by detectable deficiency of myelin in the brain, as evident on MRI. The archetypical example is Pelizaeus-Merzbacher disease (PMD) (MIM: 312080), an X-linked disease caused by mutations in the *PLP1* (MIM: 300401), which codes for proteolipid protein-1, a major component of myelin. Affected individuals can present as neonates with severe hypotonia and nystagmus or later with progressive spasticity and ataxia. Mutations in other genes, such as *SPG11* (MIM: 610844) or *FA2H* (MIM: 611026), causing spastic paraplegia, can lead to a similar phenotype, but the imaging is characteristically associated with thinning of the corpus callosum. In many inherited spastic ataxias where the affected gene is primarily involved in developmental processes, the symptoms are often most predominant and severe in children. In adults, once the myelination process has ended, the disease is less progressive and often static. Although a multitude of other genetic forms of hypomyelinating leukodystrophies have been identified in recent years, many remain uncharacterized at the gene level.^4^

In this study, we report three families with individuals affected by autosomal-recessive spastic ataxia and for whom MRI showed brain hypomyelination. In the families described here we used a combination of homozygosity mapping and exome sequencing to identify and characterize the causal variants in *NKX6-2* (MIM: 605955).

Unrelated individuals from three families were included in our study. An early-onset progressive disorder was present in all seven affected individuals. Initial and predominant features have always been motor related with variable sparing of cognitive function (Table 1). Family 1 is of North Indian descent. The two affected siblings presented with spasticity, nystagmus, and ataxia. The older sibling, individual 1 (III-1), achieved only initial motor milestones. She was able to sit up but was never able to walk or run, and she developed a complex spastic-ataxia phenotype. The initial investigations revealed cerebellar atrophy on MRI at the age of two years. The disease progressed, and at the age of 27 she is wheelchair bound and has a severe pyramidal syndrome involving predominantly the lower limbs; associated features include nystagmus, hypometric saccades, reduced up-gaze, very limited voluntary eye movements, cerebellar dysarthria, titubation, and truncal and limb ataxia with dystonic posturing and torticollis (Supplemental Movie S1). Sensory examination is normal.

**Table 1:** Description of All Phenotypes Associated with *NKX6-2* Mutations

	**Individual 1**	**Individual 2**	**Individual 3**	**Individual 4**	**Individual 5**	**Individual 6**	**Individual 7**
cDNA sequence	c.121A>T	c.121A>T	c.121A>T	c.121A>T	c.121A>T	c.487C>G	c.487C>G
Amino-acid change	p.Lys41^∗^	p.Lys41^∗^	p.Lys41^∗^	p.Lys41^∗^	p.Lys41^∗^	p.Leu163Val	p.Leu163Val
Gender (male or female)	F	M	M	F	M	F	M
Age at examination (in years)	27	23	8	44	30	6	4
Age of onset (in years)	0.3	5	0.6	1	3	0.1	0.1
Disease duration	27	18	7	43	27	6	4
Disability score	4	3	3	4	4	4	4
Symptom at onset	nystagmus	ataxia	nystagmus	nystagmus	nystagmus	nystagmus	nystagmus
Pyramidal syndrome	yes	yes	yes	yes	yes	yes	yes
Cerebellar syndrome	yes	yes	yes	yes	yes	yes	yes
Ashworth score (1–4)	3	3	2	3	2	3	3
Dystonia	cervical, limbs	cervical, limbs	limbs	cervical, limbs	absent	absent	absent
Sensation	normal	normal	normal	normal	normal	normal	normal
Eye movement	Limitation of the eye movements	Hypometric sacades, nystagmus	Hypometric sacades, nystagmus	Limitation of the eye movements	hypometric sacades, nystagmus	hypometric sacades, nystagmus	hypometric sacades, nystagmus
Cognitive function	normal	normal	normal	normal	normal	abnormal, severe global psychomotor delay	abnormal, severe global psychomotor delay
Brain MRI results	cerebellar atrophy, hypomyelination	cerebellar atrophy, hypomyelination	cerebellar atrophy, hypomyelination	cerebellar atrophy, hypomyelination, thin corpus callosum	not available	white-matter changes, hypomyelination	white-matter changes, hypomyelination

Disability score was defined as follows: 0, asymptomatic; 1, able to walk but finds it difficult to run; 2, uses one stick and/or orthosis; 3, uses two sticks or a walker; 4, unable to walk and uses a wheelchair.

**Figure 1:**
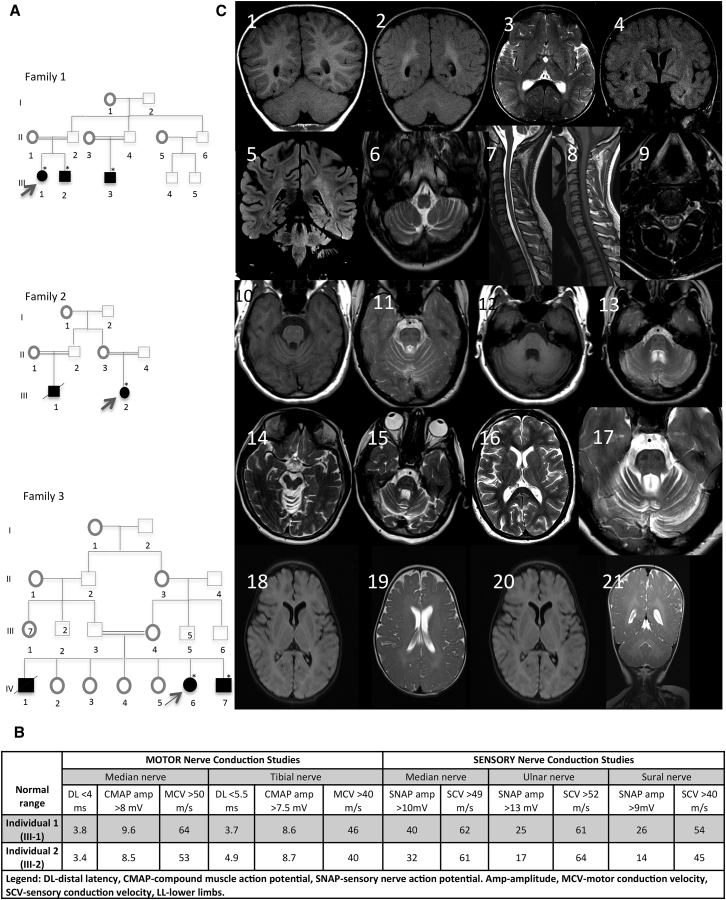
Identification of Three Families affected by Progressive Spastic Ataxia and Hypomyelination (A) Pedigree of the families with *NXK6-2* mutations. (B) Results of nerve-conduction studies showing normal myelination of peripheral nerves in family 1. (C) Magnetic resonance imaging findings in individuals with *NKX6-2* mutations. Images 1 and 2 show Coronal T1W and FLAIR sequences demonstrating periventricular FLAIR hyperintensity and corresponding iso- to hyper-intense T1W signal relative to gray matter, suggestive of hypomyelination. Some hyperintensity of the dentate hilus is noted. Images 3 and 4 show axial T2W and coronal FLAIR sequences demonstrating abnormal T2W hyper-intense signal in the periventricular white matter, globi palladi, and external capsules. Images 5 and 6 show coronal FLAIR demonstrating T2W hyperintensity in the periventricular white matter and superior cerebellar peduncles. An axial T2W sequence demonstrating hyperintensity of the inferior cerebellar penduncles is shown. Images 7–9 show sagittal T2W and T1W as well as axial T2W sequences indicating diffuse spinal-cord volume loss and abnormal T2W hyperintensity symmetrically affecting the gray matter of the ventral and dorsal horn cells. Images 10–13 show axial T1W and T2W sequences demonstrating diffuse T2W hyperintensity and T1W iso- to hypo-intensity of the pons along with relative sparing of the cortico-spinal tracts. There is disproportionate volume loss of the cerebellum as well as the superior and middle cerebellar peduncles. Images 14–17 show axial T2W. A subtle T2W hyperintense signal change affects the anterior limbs of the internal capsules, thalami, and left peritrigonal white matter. The image shows a pontine diffuse T2W hyperintense signal sparing the cortico-spinal tracts and volume loss of the cerebellum and superior cerebellar peduncles. Images 18–21 show axial and coronal T2 weighted spin echo and axial FLAIR images indicating diffusely increased T2 signal intensity in cerebral and cerebellar white matter bilaterally and symmetrically, demonstrating delayed myelination in the youngest individuals.

Her brother, individual 2 (III-2), has a similar but milder phenotype. He started walking at 12 months, but by the age of five years old he noticed difficulties with running, and at 8 years old he needed a walking frame. The disease progressed in a similar way to his sister, and he developed additional cerebellar dysarthria and ataxia while in high school. His cognitive function was within normal range; he achieved ten top GCSE school grades. He is currently 22 years old and able to walk only a few steps with maximum assistance. He has square-wave jerks and marked gaze evoked nystagmus but is able to initiate eye movements in any direction. There is retrocollis with sternocleidomastoidian muscle hypertrophy and upper-limb dystonia. In the lower limbs there is a severe pyramidal syndrome and weakness. He has significant ataxia in both upper and lower limbs. Cognitive function remains normal, and he completed a university degree in business and management with second-class honors. Nerve conduction studies were performed in both siblings and showed no evidence of demyelination of peripheral nerves (Figure 1B).

Examination of the third affected individual in this family (individual III-3) was very similar and consistent with a spastic-ataxia phenotype. His first symptoms started at the age of six months, when he presented with nystagmus and hypotonia in the limbs. Symptoms progressed and included delayed motor milestones, truncal and limb ataxia, and dysarthria. At 8 years old he was already a wheelchair user. A moderate pyramidal syndrome replaced the hypotonia as the disease progressed (Supplemental Movie S2).

The second family is of Kenyan and Tanzanian origin and has two affected siblings. Individual 4 (III-2) is currently 44 years old and the oldest in our cohort. She presented with symptoms of nystagmus, gait ataxia, and spasticity in the lower limbs at one year old. The MRI at that time confirmed cerebellar and brainstem atrophy. Currently, more than 40 years into her disease, she is wheelchair bound and presents with cervical and lower-limb dystonia, gross head titubation, hypometric saccades, horizontal nystagmus in all directions of gaze, and limited eye movements both horizontally and vertically. There is truncal and limb ataxia with severe stiffness in the lower limbs and very brisk reflexes (Supplemental Movie S3). Sensation is normal. Her affected cousin had an almost identical phenotype with a progressive spastic ataxia but died young from tuberculosis.

Children (individuals 6 [IV-6] and 7 [IV-7]) of a third consanguineous Saudi Arabian family presented with nystagmus and developmental delay, most pronounced in the motor domain. Physical examination was notable for nystagmus and hypotonia that later progressed to spasticity in the peripheries (Table 1).

MRI appearance was consistent between all seven cases (Figure 1C). The most striking finding was of hypomyelination with diffusely increased T2W and FLAIR signal in the white matter and corresponding iso- to hyper-intense signal on T1 images. Increased T2W signal was seen in the periventricular white matter, globi pallidi, external capsules, superior and inferior cerebellar peduncles, dentate hilus, and the pons. There was relative sparing of the cortico-spinal tracts in the brainstem. Cerebellar atrophy was found in adult cases, in addition to diffuse spinal cord volume loss with abnormal T2W hyperintensity of the ventral and dorsal horn cells of the spinal cord.

**Figure 2:**
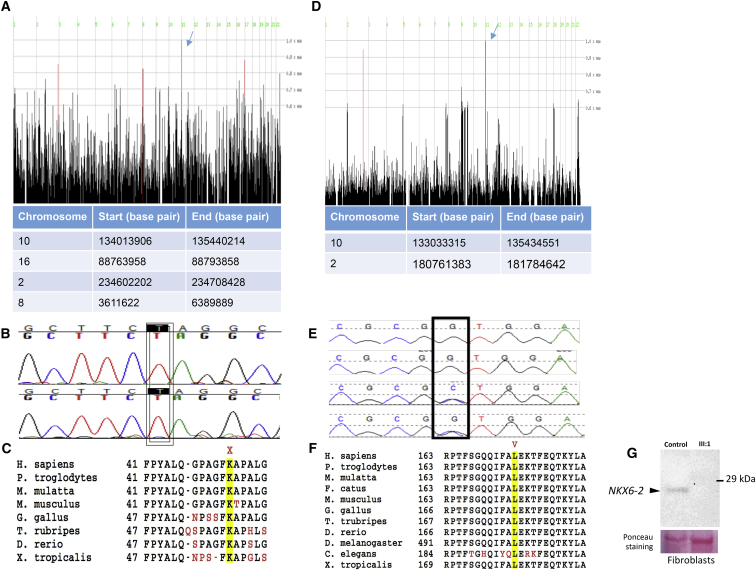
*NKX6-2* Mutations (A) Homozygosity mapping in families 1 and 2 identified four homozygous regions that were shared by the three affected individuals from families 1 and 2. The region on chromosome 10 includes *NKX6-2* (arrow) shared between the two families. (B) Sanger sequencing confirming c.121A>T in the two families. (C) Conservation of p. Lys41^∗^ across species. (D) Homozygosity mapping in family 3 identified two homozygous regions that were shared by the affected individuals. The region on chromosome 10 includes *NKX6-2* (arrow). (E) Sanger sequencing confirming c.487C>G and segregation in the family. (F) Conservation of the p.Leu163Val residue within the *NKX6-2* homeodomain across species. The mutation is marked in red above the corresponding amino acid. (G) Immunoblot analysis showing a complete absence of NKX6-2 in an affected individual (individual III-1 in family 1 in the pedigree) compared to a control individual.

The individuals included in this study were recruited along with the parents and unaffected siblings under institutional-research-board-approved research protocols (KFSHRC RAC#2060008 and UCLH 04/N034) with informed consent. DNA was extracted from peripheral blood. Extensive genetic, metabolic, and mitochondrial investigations that excluded acquired and other inherited causes were carried out for all families. Whole-exome sequencing was conducted in the three families independently and included samples from parents and from affected and unaffected children. Variants were filtered for homozygous, highly deleterious, rare mutations. After mutations were prioritized according to the above criteria, only one variant was segregating with the disease. It revealed a homozygous premature stop mutation in *NKX6-2*: c.121A>T; p.Lys41^∗^ (NM_177400.2) in families 1 and 2. There were no other variants that segregated with the disease in these two families. Although these families are not known to be related, we found that the same homozygous truncating variant in *NKX6-2* fully segregated with the disease in each family. In order to investigate whether the p.Lys41^∗^ mutation was inherited on the same haplotype, we performed homozygosity mapping in individuals from families 1 and 2. In total, we identified four regions of homozygosity that were shared by the three affected individuals; these included a 1.4 M region that contains *NKX6-2* on chromosome 10 (chr10: 134013906–35440214) (Figure 2A). Further genotype analysis confirmed the presence of a shared haplotype block surrounding *NKX6-2* (Supplementary Table S1), supporting the founder effect of the mutation. The c.121A>T mutation is located in exon 1 and creates a premature stop codon at amino acid 41. This stop codon is predicted to result in nonsense-mediated messenger RNA (mRNA) decay (NMD) and the loss of the homeobox functional domain. This mutation was not previously associated with a human phenotype and was absent in our in-house datasets of 1,284 ethnically matched exome-sequenced individuals and in public databases.^5^, ^6^

Independently, autozygosity mapping was performed in family 3. Although several autozygous intervals were found to be shared between the two affected siblings, only two were exclusive to them in comparison to the rest of the family (Chr2:180761383–181784642 and Chr10: 133033315–135434551) (Figure 2D and Figure S1). Exome sequencing was carried out in the index individual, and variants were filtered by the homozygosity coordinates as well as frequency (MAF < 0.001 when 2,363 Saudi exomes and ExAC were used^5^) and potential gene effects (splicing or coding excluding non-splicing synonymous). Only one homozygous variant remained after application of these filters: *NKX6-2* (NM_177400.2; c.487C>G; p.Leu163Val), and it fully segregated with the disease in the family under a fully penetrant autosomal-recessive model (Figure 2E). This variant was only present once in the heterozygous state in our database and is absent in ExAC.^5^ The p.Leu163Val variant replaces an absolutely conserved leucine residue in the consensus homeobox domain of the protein (Figure 2F). This residue is part of an evolutionarily constrained element (Figure S2) and is predicted to be deleterious and probably damaging by SIFT and PolyPhen, respectively. The general structure of homeodomains is helix-loop-helix-turn-helix. On the basis of sequence similarity and in-silico modeling, the p.Leu163Val mutation is predicted to lie within the first helix and to be protein destabilizing (DUET^7^ ΔΔG score: 1.418Kcal/mol). On the basis of amino acid conservation between homeodomains, the leucine residue is predicted to be highly conserved.

We established a fibroblast cell line for individual 2 to examine the effect of the truncating variant on NKX6-2 protein. Immunoblot analysis on the extracted protein revealed complete absence of the expected 29 kDa band as well as the hypothetical band representing the truncated variant, which implies that the variant is probably null (Figure 2G). The remarkably similar phenotype among individuals who are homozygous for a truncating, probably null allele and those homozygous for a missense variant suggests that the latter is a functional null allele. Taken together, our results support the notion that *NKX6-2* mutations underlie the phenotype observed in the Saudi and Indian families.

**Figure 3:**
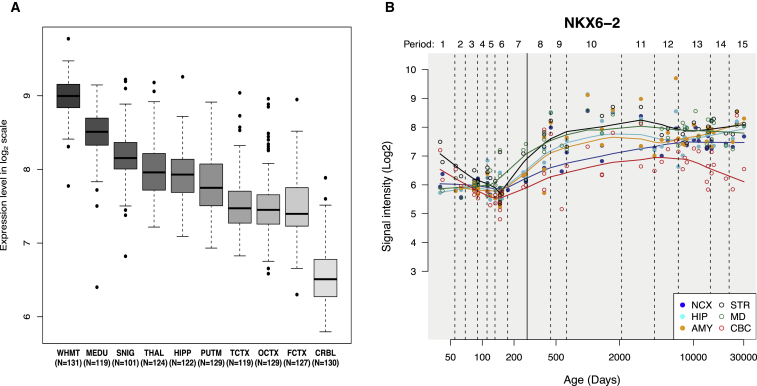
*NKX6-2* Expression in Normal Developing Brain and Adult Brain (A) *NKX6-2* expression in different brain areas in adult pathologically normal human brains.^8^*NKX6-2* is expressed in all ten brain regions; the highest expression is detected in white matter. Abbreviations are as follows: WHMT, white matter; MEDU, medulla; SNIG, substantia nigra; THAL, thalamus; HIPP, hippocampus; PUTM, putamen; TCTX, temporal cortex; OCTX, occipital cortex; FCTX, frontal cortex; and CRB, cerebellum. (B) *NKX6-2* expression in developing brain.^9^ Abbreviations are as follows: NCX, neocortex; STR, striatum; HP, hippocampus; AMY, amygdala; MD, midbrain; and CBC, cerebellum.

In humans, *NKX6-2* is known to be expressed in developing and adult human brain (Figures 3A and 3B). Although GTEx^10^ reports the highest expression in spinal cord and substantia nigra, BRAINEAC^8^ suggests that intralobular white matter expresses the most *NKX6-2* mRNA and that medulla and substantia nigra follow (Figure 3A). Because *NKX6-2* is known to be a transcription factor, we investigated these findings further in silico by using the algorithm for the reconstruction of accurate cellular networks (ARACNe) based on an adaptive partitioning (AP) strategy. In this way, we identified *NKX6-2*’s most probable target genes in human white matter (the location of maximal expression). The resulting regulon, which consisted of 407 predicted target genes, was significantly enriched for genes associated with development of the central nervous system (GO: 0007417, p value 0.0049) and cytoskeletal protein binding (GO: 0008092, p value 6.0 × 10^−4^). Interestingly, within this gene set are transcription factors known to be required for the generation of mature postmitotic oligodendrocytes, namely *NKX2-2* (MIM: 604612) and *SOX10* (MIM: 602229).^11^ Furthermore, when we used weighed genes co-expression analysis (WGCNA) to create modules of highly co-expressed genes and assessed the *NKX6-2* regulon for enrichment within these modules, we identified a significant overlap of the genes within the regulon (Fisher’s Exact test) in three co-expression modules (black, brown and salmon) all enriched for oligodendrocytes markers (p value 0.03, 7.2 × 10^−12^ and 1.13 × 10^−126^, respectively). These findings strongly suggest that in keeping with the mouse model, *NKX6-2* is a transcription factor involved in the maturation of oligodendrocytes in humans.

**Figure 4:**
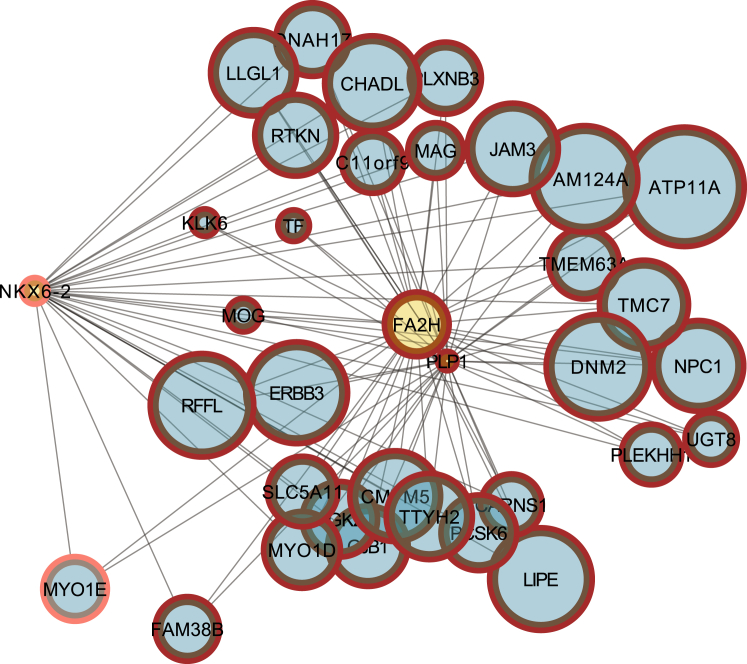
A bottom-up Plot Showing a Different Connectivity of *NKX6-2* Compared to *PLP1* and *FA2H,* Suggesting a Regulatory Role of *NKX6-2* Seed genes associated with hereditary spastic paraplegia contain an inner yellow circle, whereas context genes are represented by inner blue circles. The context genes in the plot are enriched for genes that the algorithm for the reconstruction of accurate cellular networks (ARACNe) based on an adaptive partitioning (AP) strategy predicts are found in the regulon of *NKX6-2* (23 out of 35, FET p value 2.2 × 10^−16^).

We extended our analysis further to place *NKX6-2* within the context of known Mendelian disorders. Within the *NKX6-2* regulon, we identified 72 genes that are already linked to a Mendelian disorder within the Online Mendelian Inheritance in Man (OMIM) database. Among this list we identified *FA2H* and *PLP1* (Spastic Paraplegia 35 (MIM: 612319) and Pelizaeus-Merzbacher Disease (MIM: 312080)) as well as five genes in which mutations are associated with peripheral demyelinating disorders (*PMP22* [MIM: 601097], *LITAF* [MIM: 601098], *NDRG1* [MIM: 601455], *SH3TC2* [MIM: 601596], *SOX10* [MIM: 609136], and *YARS* [MIM: 608323]) (Figure 4). Given these findings, we used the tool Phenomizer to identify a set of known Mendelian disorders with significant phenotypic similarity to individuals with bi-allelic *NKX6-2* mutations and check for enrichment of this gene set among the 72 genes and associated disorders identified within the *NKX6-2* regulon. Remarkably, we found that the *NKX6-2* regulon was significantly enriched for disorders with high phenotypic similarity to our individuals (p value 0.00079). Thus, as well as demonstrating that bi-allelic mutations in *NKX6-2* produce a hypomyelinating disorder, these findings imply that among the remaining 335 genes within the *NKX6-2* regulon, there are likely to be other gene mutations associated with disorders involving myelin generation and maintenance in the central nervous system.

*NKX6-2* is a member of the NKX protein family of transcription factors, which have established roles during the development of an organism. Awatramani et al. have demonstrated the important role of *NKX6-2* as a gene-repressor transcription factor regulating oligodendrocyte gene expression in mouse oligodendrocytes.^12^ Furthermore, they show that *NKX6-2* is working in tandem with several other genes involved in myelination,^12^ supporting our findings that *NKX6-2* acts within the same pathways of genes already associated with myelination. The homeobox is a highly conserved 180 bp DNA-binding domain that mediates specific contact with the minor and major grooves of DNA in a strictly sequence-specific fashion.^13^, ^14^, ^15^ It is likely that disrupting this sequence by replacing the highly conserved leucine residue will disrupt DNA binding. All known functions of *NKX6-2* are thought to be mediated through its transcription activity in the nucleus. We note that the equivalent residue in *NKX6-1* (p.Leu521) (MIM: 602563) is also invariant in humans, as evident from the 60,709 individuals whose sequence data are available through ExAC.^5^ It is conceivable that the cerebellar atrophy represents a more severe phenotype only seen in those with bi-allelic truncation. However, these individuals are generally much older than the two young siblings with the missense variant, so it might also represent an age-related phenotype.

*NKX6-2* was originally identified in the search for novel homeobox-domain-containing genes when the mouse embryonic cDNA library was probed.^16^ The strong expression in the postnatal glial cells and testes of mouse prompted the original name *GTX*.^16^ Although there are three members of *NKX6* (1–3), *NKX6-2* is mostly related to *NKX6-1*, with which it shares significant structure homology and overlapping domains of expression.^12^ The duo *NKX6-2* and *NKX6-1* has been extensively studied in the context of class II homeodomain transcription factors, which are induced by *SHH* (MIM: 600725) in the course of neural-tube patterning to confer ventral progenitor cell identities.^17^, ^18^ Both genes are co-expressed initially in a broad ventral domain, but their expression later becomes mutually exclusive, in part because of the repression of *NKX6-2* by *NKX6-1*.^19^ Although *NKX6-2* deficiency causes de-repression of *DBX1* and a resulting increase in V0 and decrease in V1 neurons, there was no net loss of motor neurons, and the apparent lack of a discernible phenotype in *Nkx6-2*^*−/−*^ mice was assumed to be caused by redundancy with *NKX6-1*.^19^ The redundancy model is supported by the observation that although there is significant developmental arrest of motor neurons in *Nkx6-1*^*−/−*^ mice, this arrest is compounded in double mutants.^19^, ^20^

Although the above studies were helpful in defining the location of *NKX6-2* in the developmental network during neural tube development, they suggested that its role was most likely dispensable. However, Southwood generated another knockout mouse line that provided critical insight into the indispensable role of *NKX6-2*.^21^ In addition to confirming the previously established strong expression of *NKX6-2* in the brain and spinal cord, they demonstrated a strong and long-lasting expression in oligodendrocytes well into adulthood.^21^ Importantly, loss of *NKX6-2* was found to result in significant motor and behavioral deficits. This phenotype was delayed postnatally and coincided with the timing of maximum myelination, which prompted the investigation of myelin in these mice. Significant abnormalities, including hypomyelinated and even naked axons, were noted in myelin, although they were less marked than those caused by other gene knockouts, e.g., md rats, quaking mice, and Mag-null and Cgt-null mice.^22^, ^23^, ^24^ Not only were these abnormalities seen in all examined white-matter tracts, but they also were extended to the cranial nerves, including the optic nerve.

The phenotype we observe in individuals with bi-allelic deleterious variants in NKX6-2 is supported by the previously published data on mouse and oligodendrocyte NKX6-2 deficiency^12^, ^21^, ^25^ in several aspects. Most conspicuous is the resemblance in myelin pathology, which we also note to be relatively mild in comparison to classical hypomyelinating leukodystrophies. Of particular relevance is the specific reference by Southwood et al. that the optic nerve myelin is significantly decreased given the prominent and universal nystagmus phenotype in our individuals. Nystagmus is a common feature of hypomyelinating and demyelinating disorders, and although the pathogenesis is not fully understood, it is thought to be caused by impaired visual feedback loops during development.^26^ Also relevant is the finding that motor neurons in the spinal cord develop with only subtle perturbation from their progenitors in *Nkx6-2*^−/−^ mice; this perturbation appears to be recapitulated in affected individuals who have no evidence of lower motor-neuron disease.

Furthermore, on the basis of in silico analysis, gene-regulatory networks, and co-expression data in humans, we show that *NKX6-2* is involved in the genesis and development of oligodendrocytes. These findings are mirrored in the clinical phenotype of *NKX6-2*-mutated individuals who clinically progress and then remain static after myelination is complete. Most importantly, in agreement with previous work on animal and cell models,^25^ we provide evidence that within the *NKX6-2* regulon there are other genes already linked to Mendelian disorders associated with spastic-ataxia and/or hypo or de-myelination. Apart from placing the *NKX6-2* within the myelin regulation pathway as previously reported and linking it to a human Mendelian disorder when that pathway is disrupted, our data suggest that other such genes in the *NKX6-2* regulon could be involved in central myelination and cause diseases in humans.

In conclusion, we show that the *NKX6-2* is recurrently mutated in several families and individuals with a spastic-ataxia and hypomyelinating leukodystrophy phenotype. After development and in adolescence, the phenotype stabilizes clinically. This syndrome confirms the non-redundant role of *NKX6-2* in myelin homeostasis in the central nervous system and expands the genetic causes of spastic ataxia, the heterogeneity of developmental genes, and inborn errors of myelin metabolism in humans.

## Web Resources

ExAC, http://exac.broadinstitute.org
Online Mendelian Inheritance in Man (OMIM), http://www.omim.org
Phenomizer, http://compbio.charite.de/phenomizer/

## Supplemental Data

Document S1. Supplemental Methods, Figures S1 and S2, and SYNAPSE Contributors

Table S1. Haplotype-Analysis Data in Families 1 and 2

Movie S1. Clinical Findings Related to *NKX6-2* Mutation in an Adult from Family 1 Individual III:1 from family 1 presents with ataxia, dystonia, severe lower-limb spasticity, and significant eye-movement abnormalities.

Movie S2. Clinical Findings Related to *NKX6-2* Mutation in a Child Individual III:3 from family 1. The phenotype is less severe than in the affected adults.

Movie S3. Clinical Findings Related to *NKX6-2* Mutation in an Adult from Family 2 Individual III:1 from family 2.

Document S2. Article plus Supplemental Data
